# ^18^F-FDG-Uptake in Mediastinal Lymph Nodes in Suspected Prosthetic Valve Endocarditis: Predictor or Confounder?

**DOI:** 10.3389/fcvm.2021.717774

**Published:** 2021-08-11

**Authors:** Derk ten Hove, Bhanu Sinha, Andor W. J. M. Glaudemans, Anna Gomes, Laurens E. Swart, Wilco Tanis, Ricardo P. J. Budde, Riemer H. J. A. Slart

**Affiliations:** ^1^Department of Nuclear Medicine and Molecular Imaging, University of Groningen, University Medical Center Groningen, Groningen, Netherlands; ^2^Department of Medical Microbiology and Infection Prevention, University of Groningen, University Medical Center Groningen, Groningen, Netherlands; ^3^Department of Cardiology, Maasstad Ziekenhuis, Rotterdam, Netherlands; ^4^Department of Cardiology, HagaZiekenhuis, The Hague, Netherlands; ^5^Department of Radiology and Nuclear Medicine, Erasmus University Medical Center, Rotterdam, Netherlands; ^6^Department of Biomedical Photonic Imaging, Faculty of Science and Technology, University of Twente, Enschede, Netherlands

**Keywords:** FDG (^18^F-fluorodeoxyglucose)-PET/CT, mediastinal lymph node, retrospective analysis, visual analysis, prosthetic valve endocarditis

## Abstract

**Introduction:** Prosthetic valve endocarditis (PVE) is a serious disease affecting ~0.4% of prosthetic valve recipients per year. ^18^F-FDG-PET/CT has high sensitivity and specificity for PVE and is included as major criterion for the diagnosis in recent guidelines of the European Society of Cardiology. We addressed the question whether increased FDG-uptake in mediastinal lymph nodes could help to support the visual diagnostic assessment of PVE.

**Methods:** In this sub-analysis of a previously published retrospective multicentre study, 160 unique patients were identified who underwent ^18^F-FDG-PET/CT for evaluation of suspected PVE. ^18^F-FDG-PET/CT was performed in adherence to the European Association of Nuclear Medicine guidelines of 2015 and scans were assessed for signs of mediastinal lymph node activity by 2 experienced nuclear medicine physicians who were blinded to clinical context. Clinical diagnosis of PVE had been established based on surgical findings or multidisciplinary consensus after a 1-year follow-up in 80 of 160 patients (50%).

**Results:** In total, 52 patients showed increased mediastinal lymph node activity. Mediastinal lymph node activity on ^18^F-FDG-PET/CT did not increase diagnostic accuracy when added to the visual analysis of scans for signs of PVE: *X*^2^: 0.118, *p* = 0.731). After excluding patients with known confounders for ^18^F-FDG-PET/CT, namely use of Bioglue® during prosthetic valve implantation and C-reactive protein levels below 40 mg/L, mediastinal lymph node activity was still not of additional diagnostic value compared to visual analysis alone (X^2^:0.129, *p* = 0.723).

**Discussion:** Assessment of mediastinal lymph node activity did not improve ^18^F-FDG-PET/CT diagnostic accuracy for suspected PVE compared to visual assessment of the valve alone, as it seems to be a rather a specific finding, that might be caused by sternal wound or mediastinal infections or even by subclinical respiratory infections. Future studies might elucidate whether increased FDG active lymph nodes indicate a high-risk patient group and whether more detailed assessment of mediastinal lymph nodes could improve their additional diagnostic benefit.

## Introduction

Prosthetic valve endocarditis (PVE) is a serious condition with a 1-year mortality of up to 50% (1) and affecting 4–6 per 1,000 patients with prosthetic heart valves per year (2–4). The diagnosis is challenging because the clinical presentation of PVE is variable and because one of the cornerstones for the diagnosis, echocardiography, severely suffers from prosthetic material-related artifacts, limiting its accuracy (5). At the same time, timely accurate diagnosis and treatment are vitally important to minimize the risk of life- threatening complications and the need for re-surgery. The modified Duke criteria have long been used for the diagnosis of infective endocarditis (IE) (6). In 2015, both the American Heart Association (AHA) (7) and the European Society of cardiology (ESC) included additional imaging modalities in their guidelines, the latter included them even as formal major criteria for IE, leading to an updated modified ESC diagnostic scoring system (8). One of these additional imaging modalities is ^18^F-Fluorodeoxyglucose positron emission tomography (^18^F-FDG-PET/CT). Recently, the AHA recommended using ^18^F-FDG-PET/CT for cases of “possible” IE, because of its ability to reclassify up to 76% of these cases to “definite” IE (9, 10) while it can also help establish alternative sources of infection supporting rejection of IE (11).

The most recent meta-analysis evaluating the diagnostic value of ^18^F-FDG-PET/CT for diagnosing PVE in particular found a pooled sensitivity and specificity of 86 and 84%, respectively (12). This is in line with an earlier comprehensive systematic review that found a range for sensitivity and specificity between 71–100 and 73–100%, respectively (13). Recently, an extensive guideline on the acquisition and interpretation of ^18^F-FDG-PET/CT for IE was published (14), which included criteria to be included in the evaluation of suspected PVE, such as the location of a lesion relative to the suspected heart valve, lesion intensity and heterogeneity of FDG uptake. An open question regarding ^18^F-FDG-PET/CT interpretation, however, is whether other indicators exist that could be used to enhance ^18^F-FDG-PET/CT diagnostic accuracy for endocarditis. A recent study for instance evaluated bone marrow or spleen hypermetabolism for this indication with promising results (15). We evaluated whether the presence of FDG-active mediastinal lymph nodes could be used to confirm or improve the diagnosis of PVE. Since all infections can lead to regional lymphadenopathy and information about mediastinal lymph nodes is already provided when performing ^18^F-FDG-PET/CT, incorporating these findings into the analysis and interpretation of the ^18^F-FDG-PET/CT scan would be a relatively simple method to increase its diagnostic accuracy. This article aims to evaluate whether combining mediastinal lymph node FDG activity in the evaluation of suspected PVE using ^18^F-FDG-PET/CT may be of additional value.

## Methods

### Study Population

In this retrospective multicentre study, patients from six cardiothoracic centers in the Netherlands were included if they had >1 prosthetic heart valve(s) and had undergone ^18^F-FDG-PET/CT imaging for suspicion of PVE. Part of these data have previously been published (16). The study was approved and informed consent was waived by local Medical Ethics Committees (registration nrs. EMC: MEC-2015-172, UMCG: METc2015/033). Clinical follow-up for all patients was at least 1 year. Demographic data of the included patients were recorded for analysis, together with relevant clinical data and technical data of the included scans. In case patients had multiple episodes of suspected PVE, only the first scan acquired for suspicion of PVE was included to avoid confounding by previous or ongoing episodes of PVE.

### FDG-PET/CT Acquisition and Assessment

Patient preparation for the ^18^F-FDG-PET/CT scan was documented. This included adherence (or non-adherence) to a minimum of 6 h fasting period, a 24 h high-fat low carbohydrate (HFLC) diet and use of a heparin IV injection 15 min prior to FDG administration (14). ^18^F-FDG-PET/CT was performed in adherence to European Association of Nuclear medicine (EANM) guidelines (17). EANM Research Ltd (EARL) standardized reconstructions were available in 116 (73%) of scans. ^18^F-FDG-PET/CT scans were evaluated visually in the Syngo.via VB30 software system [Siemens Healthcare GmbH ©]. All obtained scans were assessed by 2 experienced nuclear medicine physicians (AWJMG and RS) who were blinded to the patients' clinical context. First, it was visually determined whether there was PVE or not, based on several criteria (pattern and intensity of uptake, persistent uptake at non-attenuated images). Cases of disagreement were resolved through consensus reading and this led to a binary score of infection being either present or not based on the ^18^F-FDG-PET/CT scan (16). Subsequently, the presence of increased FDG-uptake in mediastinal lymph nodes was evaluated. Increased uptake was defined as uptake higher than the average FDG activity in the surrounding mediastinum and with presence of lymph nodes at the same location at the low dose CT scan.

### Reference Standard for Prosthetic Valve Endocarditis

As previously reported, if surgery had been performed, macroscopic evidence of infection during surgery and findings during subsequent histopathology and/or cultures of the removed tissues consistent with endocarditis were considered the standard for diagnosis of PVE. If surgery was not performed, the diagnosis was established through multidisciplinary consensus of physicians from the Departments of Cardiology/Thoracic Surgery, Infectious Diseases, Medical Microbiology, Radiology, and Nuclear Medicine (the Endocarditis Team) using all available clinical data including patient outcomes over a minimum follow-up of 1 year, to avoid reliance on ^18^F-FDG-PET/CT scan results for the diagnosis as much as possible (16).

### Statistics

Demographic data were retrieved as either mean with standard deviation for normally distributed variables or median with interquartile range for the non-normally distributed data. Absolute numbers with percentages were used for frequencies. Comparison between groups was performed using the Student's *T*-test for normally distributed data, Mann-Whitney's *U*-test for non-parametric data and Fisher's exact test for categorical data. Logistic regression was used to evaluate the predictive value of ^18^F-FDG-PET/CT and the additional value of mediastinal lymph nodes. *P* < 0.05 were considered statistically significant. All analyses were performed using IBM SPPSS 26 [IBM Corp].

## Results

### Patient Characteristics

As previously reported, 160 ^18^F-FDG-PET/CT scans, acquired for suspicion of PVE, were analyzed. The suspicion of PVE was confirmed in 80 patients on final diagnosis, while the diagnosis was rejected in the remaining 80. An overview of the demographic data, grouped by presence or absence of increased mediastinal lymph node activity, is presented in Table 1. Of the 160 included patients, 108 (67.5%) were male, and the median age of included patients was 62 years old. Their average BMI was 25.2 and 33 had previous episode(s) of infective endocarditis. Diabetes mellitus was present in 23 (14.4%) patients and the surgical adhesive Bioglue had been used during implantation of the prosthetic heart valve in 4 patients. There were no statistically significant differences in the demographic data between patients with normal and elevated FDG activity in mediastinal lymph nodes.

**Table 1:** Demographic data.

	**Suspected prosthetic valve endocarditis (** ***n*** **=** **160)**			***p***
	**Lymph node negative**	**Lymph node positive**	**Total**	
**Demographics**	*n* = 108	*n* = 52	*n* = 160	
Age, median (IQR)	65 (45–73)	61 (43–74)	62 (43–73)	0.78
Male, n (%)	69 (64%)	39 (75%)	108 (8%)	0.21
BMI, mean (SD)	25.4 (4.9)	25.0 (5.3)	25.2 (5.0)	0.68
Diabetes mellitus, n (%)	19 (18%)	4 (8%)	23 (14%)	0.15
Prior endocarditis, n (%)	21 (19%)	12 (23%)	33 (21%)	0.54
**Primary valve location, n (%)**				
Aortic	88 (82%)	44 (85%)	132 (83%)	0.50
Mitral	8 (7%)	4 (8%)	12 (8%)	
Pulmonic	11 (10%)	3 (6%)	14 (9%)	
Tricuspid	1 (1%)	1 (2%)	2 (1%)	
**Modified duke criteria [Li, 2000], n (%)**				
PVE rejected	43 (40%)	14 (27%)	57 (36%)	0.09
Possible PVE	32 (29.6%)	13 (25%)	45 (28%)	
Definite PVE	33 (31%)	25 (48%)	58 (36%)	
Surgical/histopathological confirmation	22 (20%)	18 (35%)	40 (25%)	0.13
**Final diagnosis PVE**				
Confirmed	50 (46%)	30 (58%)	80 (50%)	0.24
Rejected	58 (54%)	22 (42%)	80 (50%)	
**Confounders**				
Antibiotic treatment duration (days), median (IQR) *n = 103*	10 (6–16)	13 (6–27)	11 (6–20)	0.30
CRP (mg/L), median (IQR) *n = 151*	53 (18–90)	57 (28–123)	54 (20–96)	0.29
Leucocytes (×10^9^/L), median (IQR) *n=98*	9.6 (7.5–11.6)	8.3 (5.7–11.1)	9.2 (7.3–11.2)	0.23
Use of bioglue, n (%)	3 (3%)	1 (2%)	4 (3%)	1.00
Time from implantation (days), median (IQR) *n = 157*	663 (74–2,216)	730 (88–2,211)	685 (82–2,216)	0.75
**Outcomes**				
Mortality, n (%)	9 (8%)	10 (19%)	19 (12%)	0.07

### Acquisition Parameters

Patient preparation included a minimum 6 h fasting period for all patients. An HFLC diet, starting 24 h before the scan, was used in 88 patients (55%), while a Heparin injection (50 IU/kg) 15 min before FDG administration was given to 20 patients (12%). Complete myocardial suppression, defined as myocardial uptake equal to or less than thoracic blood pool activity, was achieved in 91 patients (56.8%).

### Visual Assessment of PVE

As previously reported, visual assessment of the ^18^F-FDG-PET/CT scan predicted the presence of PVE by logistic regression (*X*^2^: 81.4; *p* < 0.001; B: 3.545; *p* < 0.001). The corresponding sensitivity and specificity were 74 and 91%, respectively (16). Excluding patients with known confounders, such as a C-reactive protein <40 mg/L and prior use of surgical adhesives, led to an increased sensitivity and specificity of 91 and 95%, respectively (16).

### Mediastinal Lymph Nodes

**Figure 1:**
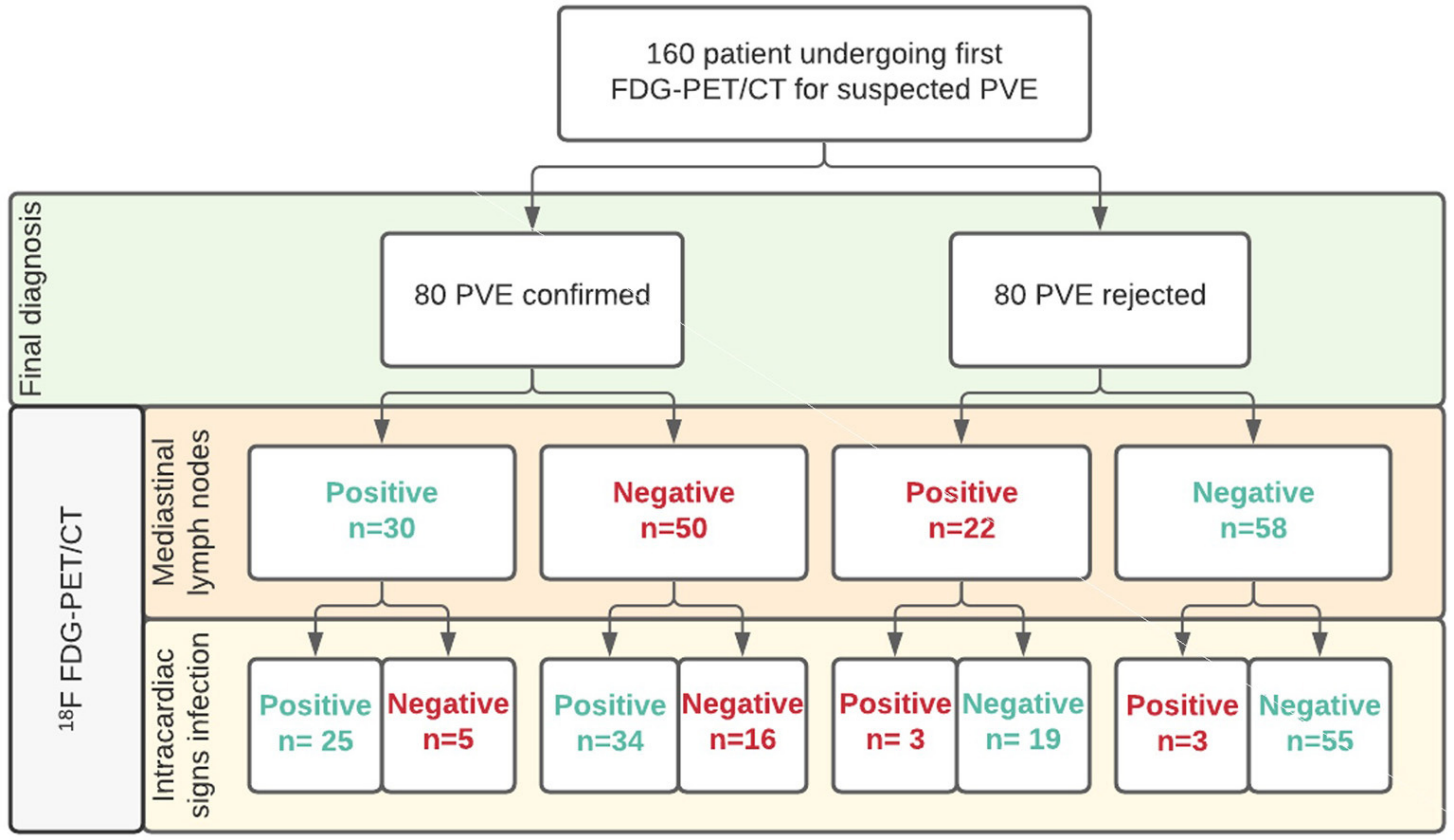
Overview of patient groups according to clinical and PET/CT results for mediastinal lymph nodes in diagnosing PVE. Legend: Correct classifications in green, misclassifications in red.

**Figure 2:**
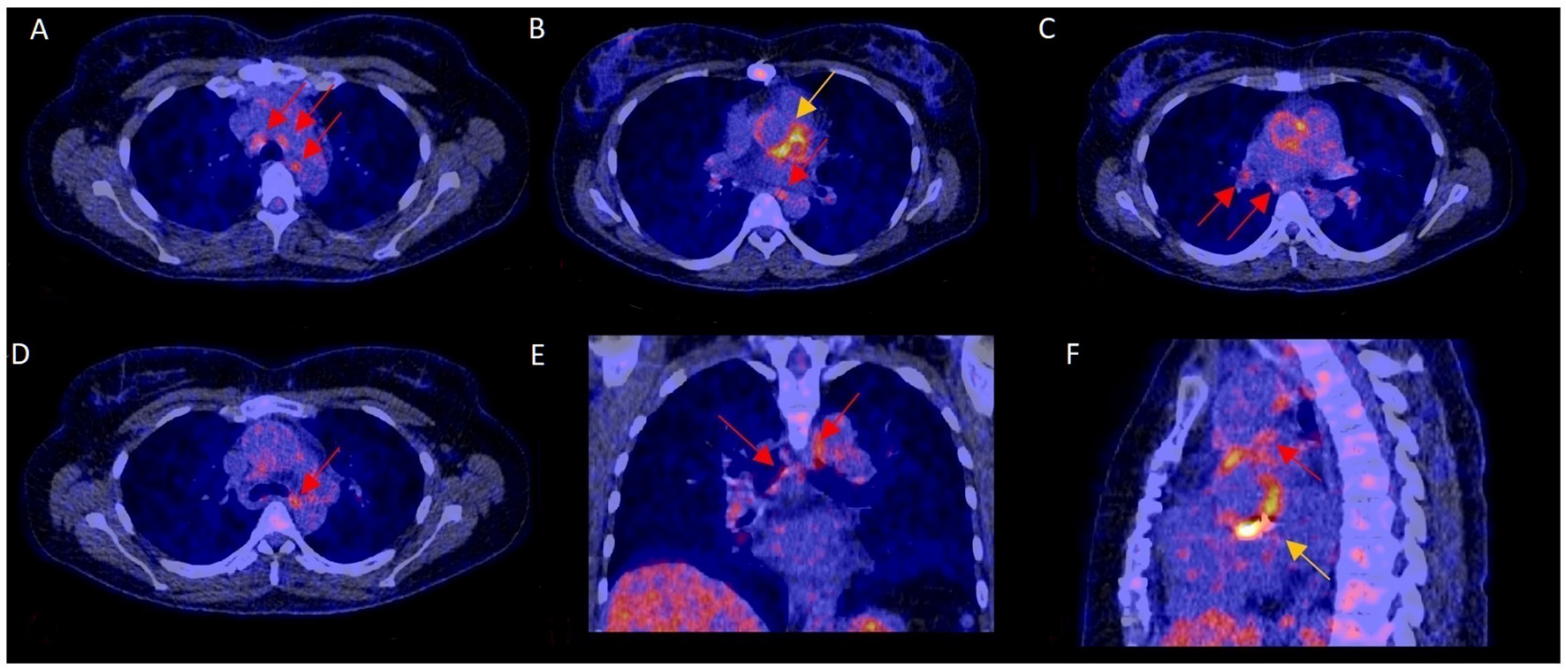
^18^F-FDG-PET/CT scan with increased mediastinal lymph node FDG activity and confirmed PVE. In this 40–45 year-old female with blood culture-negative PVE, FDG uptake was markedly increased in mediastinal lymph nodes (**A–F**: red arrows) and the aortic prosthetic valve (**B,F**: yellow arrows). Surgery showed aortic valve damage consistent with infection, confirming PVE. The pathogen could not be identified by either culture or 16s PCR.

Increased FDG uptake in mediastinal lymph nodes was present in 52 (32%) patients, but correlated poorly with the final diagnosis, as only 30 of these cases represented PVE while the remaining 22 had no PVE at final diagnosis. In the remaining 108 (68%) patients no elevated FDG activity was observed in their mediastinal lymph nodes. This also did not predict either presence or absence of PVE, as 50 patients in this group were diagnosed with PVE, while 58 were not (see Figure 1). Adding increased mediastinal lymph node FDG activity to the logistic regression model did not significantly improve visual analysis of PVE: *X*^2^: 0.118; *p* = 0.731. Patients with increased FDG uptake in mediastinal lymph nodes were slightly more likely to have a positive diagnosis of PVE and less likely to have a rejected or possible diagnosis of PVE by the Modified Duke criteria (6), but this was a trend only (*p* = 0.09, see Table 1). An example of PVE with lymph node involvement is shown in Figure 2.

Including only patients with C-reactive protein level above 40 mg/L and no prior use of surgical adhesives did not significantly improve visual analysis of PVE based on increased mediastinal lymph node activity (*X*^2^: 0.129, *p* = 0.723). Increased FDG activity was present in mediastinal lymph nodes in 31 of 89 patients (35%). This matched with PVE in only 20 of the 31 patients. Likewise, of the 58 patients without elevated FDG activity in their mediastinal lymph nodes, PVE was confirmed in 26 and rejected in 32.

**Figure 3:**
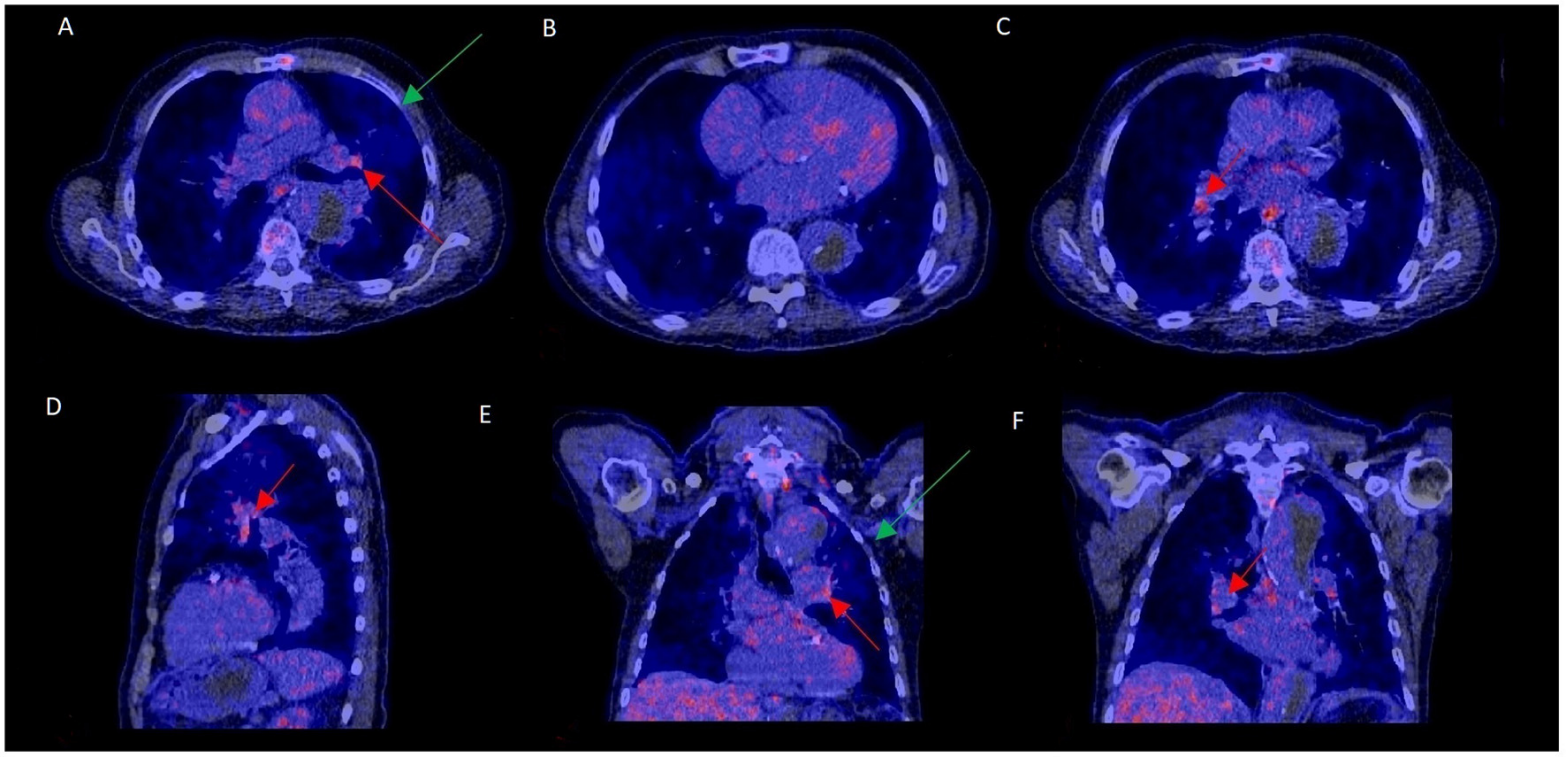
^18^F-FDG-PET/CT scan with increased mediastinal lymph node FDG activity without PVE. This 70–75 year old patient presented with a type B aortic dissection and an unexplained fever. PET/CT was performed to rule out PVE. FDG uptake was increased in mediastinal hilar lymph nodes (**A,C–F**: red arrows) but not at the aortic prosthetic valve **(B)**. PVE was ruled out during surgery, performed to treat the dissection. Slightly elevated FDG uptake and a consolidation in the left upper lobe (**A,E**: green arrows) were consistent with a mild pneumonia in remission, which may have accounted for the increased lymph node activity.

Increased mediastinal lymph node activity without a final PVE diagnosis could be explained by a concurrent LVAD infection in 1 patient and in another one due to a pneumonia in remission (Figure 3). In the remaining 20 cases, the underlying cause of false positivity could not be established. The time interval after prosthetic valve implantation was compared for those with and without increased lymph node activity to rule out that uptake in lymph nodes was caused by postoperative sterile inflammation. The median interval between valve implantation and ^18^F-FDG-PET/CT was similar for both groups, with a median of 95 weeks for those with normal mediastinal lymph node activity and 104 weeks for those with increased mediastinal lymph node activity (*p* = 0.75). Extracardiac activity on ^18^F-FDG-PET/CT was documented for brain, lungs, sternum, mediastinum, spine, spleen, liver and kidneys as these were regarded as potential foci of disseminated disease. However, none of these showed a statistically significant correlation with mediastinal lymph node activity. Only the total number of extracardiac foci showed a trend: *p* = 0.08. No relationship was found between duration of antibiotic use and presence of mediastinal lymph node activity (*p* = 0.30).

### Prognosis

Of the 52 (32%) patients with increased mediastinal lymph node FDG activity, 10 (19%) died during the 1-year follow-up. In the group with normal mediastinal lymph node activity, 9 of 108 (8%) of patients died. This difference showed a trend, *p* = 0.07 (hazard ratio of 2.62 [95% CI 0.99–6.91]).

## Discussion

In this study, we evaluated the additional value of assessment of mediastinal lymph nodes on ^18^F-FDG-PET/CT as an interpretation criterion for the diagnosis of PVE. Our main findings were that increased FDG uptake in mediastinal lymph nodes was not indicative of PVE, and adding an assessment of mediastinal lymph nodes to the visual interpretation of the scans did not result in increased diagnostic accuracy of ^18^F-FDG-PET/CT for PVE.

Non-active mediastinal lymph nodes were frequently observed in patients diagnosed with PVE. An explanation could be that implanted heart valves are avascular and even in native heart valves lymph ducts are scarce. In a study published in 1977, presence of lymph ducts could not be demonstrated in aortic or pulmonic valves, even with stereomicroscopy (18). Micro-organisms can also evade the immune system through biofilm formation in endocarditis (19), which together could explain a limited, or even absent localized immune response, particular in the early stages of infection or in vegetations that are limited to the valve leaflets.

Elevated mediastinal lymph node FDG activity was not indicative of PVE either. While this was unexpected, the reasons for this finding could be multicausal. Increased lymph node activity in some cases might also have been caused by sternal wound infections and/or mediastinitis, or even simply a concomitant respiratory tract infection, as even subclinical respiratory infections may lead to increased FDG uptake in mediastinal lymph nodes (20). Furthermore, after thoracic surgery is performed, regeneration and healing lead to increased FDG uptake in the area and mediastinal lymph nodes could become involved in this reaction. However, an argument against this latter explanation is that the median interval between prosthetic valve implantation and ^18^F-FDG-PET/CT was similar for patients with and without increased mediastinal lymph node activity.

Assessment of mediastinal lymph node activity in this study was performed using a binary score of either normal or increased FDG uptake. The exact drainage route of heart valves is not exactly known, neither is it known whether there is a difference between one or more positive lymph nodes or the amount of FDG uptake in the lymph nodes. Finding multiple lymph nodes with increased FDG uptake may increase its added value for PVE and the same could be true for the exact location of the involved lymph nodes. According to anatomical findings, the cardiac lymph ducts converge into one supracardiac channel that runs from the aortic root toward either the right thoracic duct or the right angulus venosus in the most common (~80%) anatomical variant (18). Since lymph tracts are one-way vessels, it is reasonable that only lymph nodes along this precise tract are related with PVE, with the lymph nodes proximal to the aortic root showing increased FDG uptake first.

Another remarkable finding in our study was that increased mediastinal lymph node activity might be associated with poor patient outcome. A possible explanation of this could be that in patients with PVE mediastinal lymph node activity increases only late, and finding increased lymph node activity therefore indicates more advanced disease. As this finding was a statistical trend only, it would need to be validated in future studies before any definitive conclusions can be drawn regarding the role of mediastinal lymph nodes for the prognosis of PVE.

### Limitations

Limitations of this study include its retrospective design and the availability of ^18^F-FDG-PET/CT findings to the expert team determining the final diagnosis. However, we strongly tried to limit the risk of incorporation bias through a staged unblinding of the scan results. Findings during surgery and subsequent histopathology, cultures and 16S PCR of removed tissues were used as the gold standard whenever surgery had been performed. In case surgery was not performed, all other clinical information combined with a minimum of 1 year follow-up was used as a composite gold standard for the diagnosis.

In our cohort, patient preparation using an HFLC diet and heparin was performed in 55 and 12% respectively, since the majority of literature about their potential benefits for ^18^F-FDG-PET/CT interpretability came after patient inclusion had begun (21–24). This may have impacted overall ^18^F-FDG-PET/CT diagnostic performance, even though the sensitivity and specificity that were found were both high. Additionally, information about an alternative diagnosis in cases where PVE had been rejected was not always available and this could have helped to better understand the underlying cause whenever the presence of increased FDG activity in mediastinal lymph node did not match with the presence of PVE. Extracardiac FDG uptake was evaluated but none of the evaluated locations showed a significant correlation with mediastinal lymph node activity.

## Conclusion

^18^F-FDG-PET/CT is a valuable tool for the evaluation of PVE, however increased FDG uptake in mediastinal lymph nodes was not indicative for presence or absence of PVE. Future prospective studies with standardized patient preparation are needed to evaluate whether increased mediastinal lymph node activity can identify patients at higher risk of unfavorable outcome and whether more detailed information about FDG active lymph nodes, such as the number of lymph nodes with increased FDG activity and precise regional location to the heart, might improve their additional diagnostic value.

## Data Availability Statement

The data analyzed in this study is subject to the following licenses/restrictions: The data are exclusively available for the purpose of reproducing the study results. Requests to access these datasets should be directed to https://zenodo.org/record/1208421/accessrequest.

## Ethics Statement

The studies involving human participants were reviewed and approved by Medical Ethics Review committee of University Medical Center Groningen. Written informed consent for participation was not required for this study in accordance with the national legislation and the institutional requirements. Written informed consent was not obtained from the individual(s) for the publication of any potentially identifiable images or data and specific care was taken to avoid including any identifiable patient information in the article.

## Author Contributions

DH wrote the article under supervision and with support of AGl, RS, BS, LS, AGo, WT, and RB. All authors discussed the results and revised the final manuscript.

## Conflict of Interest

This study was supported in part by PUSH, a collaborative framework project of the University Medical Center Groningen and Siemens Healthineers. The funder was not involved in the study design, collection, analysis, interpretation of data, the writing of this article or the decision to submit it for publication. The remaining authors declare that the research was conducted in the absence of any commercial or financial relationships that could be construed as a potential conflict of interest.

## Publisher's Note

All claims expressed in this article are solely those of the authors and do not necessarily represent those of their affiliated organizations, or those of the publisher, the editors and the reviewers. Any product that may be evaluated in this article, or claim that may be made by its manufacturer, is not guaranteed or endorsed by the publisher.
